# A randomized, double-blind, placebo-controlled multicenter study for evaluating the effects of fixed-dose combinations of vitamin C, vitamin E, lysozyme, and carbazochrome on gingival inflammation in chronic periodontitis patients

**DOI:** 10.1186/s12903-019-0728-2

**Published:** 2019-03-07

**Authors:** Ji-Youn Hong, Jung-Seok Lee, Seong-Ho Choi, Hyun-Seung Shin, Jung-Chul Park, Seung-Il Shin, Jong-Hyuk Chung

**Affiliations:** 1 Department of Periodontology, Periodontal-Implant Clinical Research Institute, School of Dentistry, Kyung Hee University, 26 Kyungheedae-ro, Dongdaemun-gu, Seoul, 02447 South Korea; 2 Department of Periodontology, College of Dentistry, Yonsei University, Seoul, South Korea; 3 Department of Periodontology, College of Dentistry, Dankook University, Cheonan, South Korea

**Keywords:** Vitamin C, Vitamin E, Lysozyme, Carbazochrome, Periodontitis

## Abstract

**Background:**

To evaluate gingival inflammation from fixed-dose combinations of vitamin C, vitamin E, lysozyme and carbazochrome (CELC) in the treatment of chronic periodontitis following scaling and root planing.

**Methods:**

One hundred patients were randomly assigned to receive CELC (test) or placebo (control) for the first 4 weeks at a 1:1 ratio, and both groups received CELC for the remaining 4 weeks. Primary outcome was the mean change in the gingival index (GI) after 4 weeks. Secondary outcomes included mean change in GI after 8 weeks and plaque index, probing depth, clinical attachment level, and VAS at 4 weeks and 8 weeks.

**Results:**

Ninety-three patients completed the study. The GI in the test group significantly decreased after 4 weeks (*p* < 0.001) and 8 weeks (*p* < 0.001). The mean change from baseline in GI significantly decreased in the test group compared to the control group after 4 weeks (*p* = 0.015). In the GEE model adjusting for age, gender and visits, the test group showed 2.5 times GI improvement compared to the control group (*p* = 0.022).

**Conclusions:**

Within the study, CELC showed a significant reduction in gingival inflammation compared with a placebo. Other parameters, however, were similar between groups.

**Trial registration:**

KCT0001366 (Clinical Research Information Service, Republic of Korea) and 29 Jan 2015, retrospectively registered.

## Background

Periodontitis is a host immunoinflammatory response induced by microbial challenge to the oral biofilm and a subsequent destruction of bone and connective tissue primarily caused by activated biological mechanisms such as matrix metalloproteinase, interleukin-1 (IL-1), and prostaglandins [1, 2]. The progression and clinical expression of the disease reveals a complexity corresponding to the net integration of the host response and susceptibility to the disease modified by environmental and acquired risk factors [3]. In the biological process of inflammation, several mediators, including pro-inflammatory cytokines, arachidonic acid metabolites, and reactive oxygen species (ROS), are involved in the pathogenesis, for which pharmaceutical inhibition has been suggested as an adjunctive approach for periodontal treatment [4, 5].

Widely known categories of host-modulating agents are represented by antiproteinases such as subantimicrobial doxycycline, nonsteroidal anti-inflammatory drugs, and bone-sparing antiresorptive agents such as bisphosphonates [6, 7]. However, there have been various ongoing trials to search for new strategies and agents to control the inflammatory process [5]. In terms of nutritional intervention for host response modulation, it has been speculated that micronutrients, including vitamins (C, E, A, and D), carotenoids, and polyphenols, downregulate pro-inflammatory cascades by acting as antioxidants to lessen oxidative stress [8, 9].

Oxidative stress, which is explained as a shifted balance towards oxidant load over antioxidative capacity, plays a key role in inflammatory tissue destruction that directly results from excess ROS generated by hyperactive phagocytic lymphocytes (e.g., polymorphonuclear leukocytes) or indirectly from the activation of redox-sensitive transcription factors, nuclear factor kappa B (NF-κB), and activating protein-1 (AP-1) to stimulate pro-inflammatory conditions during host and microbial interaction [10]. Tissue breakdown by ROS includes cell membrane lysis, DNA damage, and the degradation of collagen and extracellular matrix components, such as hyaluronic acid and proteoglycan, with the activation of proteolytic enzymes. Vitamins C and E are representative nonenzymatic antioxidants exogenously obtained through dietary intake [9, 11]. Although human clinical studies are limited, the inverse relationship of vitamins C and E to periodontal disease and their effects on immune function and anti-inflammatory properties have been reported [12–14].

The pharmaceutical evaluated in this study was a fixed-dose combination of vitamin C, vitamin E, lysozyme, and carbazochrome (CELC) at 150 mg, 10 mg, 30 mg, and 2 mg, respectively. Along with vitamins C and E, a host protective protein, lysozyme, was included to act as an antimicrobial agent through lysis of the bacterial peptidoglycan layer, inhibition of bacterial glucose uptake and acid production [15, 16]. Lastly, carbazochrome, which is used as a hemostatic drug in the medical field, was added to improve gingival bleeding. This addition is because the agent has been reported to reduce vascular hyperpermeability induced by vasoactive agents, such as thrombin, bradykinin, and histamine, resulting from the inflammatory response [17]. However, there has been a lack of clinical data on the use of carbazochrome in periodontal treatment, and evidence is needed to support the adjunctive supplemental intervention of the fixed-dose combinations using well-designed clinical trials.

The aim of this study was to evaluate the efficacy of CELC on gingival inflammation and other changes in periodontal parameters compared to the control group.

## Materials and methods

### Design of the clinical trial

This multicenter study was designed as a double-blind, randomized, controlled prospective trial to evaluate the efficacy of CELC (IGATAN F®, Myung-In, Seoul, Korea) on gingival inflammation in chronic periodontitis patients after 8 weeks of administration. The study adheres to the CONSORT guidelines and was conducted in accordance with the World Medical Association Helsinki Declaration (Version 2008). The study protocol was approved by each of the involved Institutional Review Boards (IRB) at Kyung Hee University Dental Hospital (KHDIRB1409–2), Yonsei University Hospital (2014–0074), and Dankook University Hospital (H-1411/011/003). All the participants were informed of the objectives, interventions, and possible risks and benefits of the study prior to enrollment, and written consent was obtained.

### Participants

A total of 112 patients who visited the Department of Periodontology in the dental hospitals of Kyung Hee (55 patients), Yonsei (43 patients), and Dankook Universities (14 patients) between October 2014 and May 2015 were screened for eligibility, and 100 patients were enrolled in the study. Inclusion and exclusion criteria were as follows.

Inclusion criteria:

• Aged between 19 and 80

• More than 20 natural teeth present in the oral cavity

• PD of 4–6 mm in at least one site per quadrant

• Diagnosis of generalized chronic incipient to moderate periodontitis

Exclusion criteria:

• Severe periodontal disease and need for emergency treatment or periodontal surgery consecutively

• History of hypersensitivity to the agents in the test medication

• Compromising systemic diseases that were not controlled

• Use of antiplatelet or anticoagulant agents that might induce bleeding tendency

• Use of medications that could affect the condition and healing of periodontal tissues

• History of taking antibiotics and NSAIDs for more than 3 days within 1 month

• History of any dental treatment including scaling within 1 month or any periodontal treatment except for plaque control within 6 months

• Having fixed or removable orthodontic appliances

• Being a woman who is pregnant or lactating

The total sample size was 100 patients, and they were randomly assigned to the control group and test group at a 1:1 ratio considering possible loss of 30%, which satisfied at least 35 patients in each group for the purpose of an exploratory clinical trial to investigate the clinical efficacy using the gingival index. One hundred patients who met the criteria and agreed to participate in the trial were randomly assigned to either the control or test group. The subjects were allocated according to an off-site computer-generated list (SPSS® 12.0, SPSS Inc., Chicago, IL, USA) with a stratified block randomization method using the treatment center as a stratum. The randomization assignment list was blinded to all examiners and participants, except for the person who packed and labeled the medication according to the list.

### Interventions

At the screening visit, demographic information and medical and smoking histories were collected, and a periodontal examination including the gingival index (GI, Löe & Silness 1963), plaque index (PI, Silness & Löe 1964), probing depth (PD), gingival recession/enlargement (GR/GE), and clinical attachment level (CAL) were recorded. Full mouth scaling and root planing (SRP) and oral hygiene instruction using the same toothbrush and toothpaste was prepared for the subjects enrolled in the trial. A baseline visit was performed after 4 weeks of a run-in period, and patients received either CELC or placebo. In the test group, the CELC was provided for 8 weeks. The control group received a placebo, which was composed of anhydrous dibasic calcium phosphate (CaHPO_4_), delivered in the same shape and color as the CELC for the first 4 weeks and the CELC for the last 4 weeks in the 8-week study period.

Outcome measurements included the periodontal parameters of GI, PI, PD, and CAL, and the visual analogue scale (0–100 mm VAS) was used to score the patients’ subjective reports of discomfort, bleeding and swelling in the gingiva (0 for no pain or discomfort to 10 for intense pain or discomfort). Ramfjord teeth (#16, #21, #24, #36, #41, and #44 by the F.D.I tooth numbering system) were set up to assess the efficacy and were replaced by the tooth in the adjacent or symmetrical position when a missing tooth was found. Measurements were performed at four (GI and PI) or six sites (PD and CAL) per tooth using a periodontal probe (UNC-15, Hu-Friedy, Chicago, Il, USA), and the data from the baseline, 4-week, and 8-week visits were collected. The primary outcome was the mean change from baseline in GI after 4 weeks of treatment. Secondary outcomes were to compare the mean change in GI from the baseline to 8 weeks and in plaque index (PI), probing depth (PD), clinical attachment level (CAL) and 100 mm VAS at 4 weeks and 8 weeks between the groups and within each group.

### Safety

The safety analysis included all the subjects who were randomly treated with either placebo or CELC. For the full analysis set (FAS), there were 48 patients in the test group and 49 patients in the control group. Treatment-emergent adverse events (TEAEs) were screened before and throughout the study period. The number and proportion of TEAEs were recorded in accordance with the summarized events in the Medical Dictionary for Regulatory Activities System Organ Class and Preferred Term regardless of potential causal relationships.

### Statistical analysis

The data from 48 patients in the test group and 45 patients in the control group who completely followed the protocol (PP, per protocol) were analyzed using SPSS 12.0 (SPSS Inc., Chicago, IL, USA). Descriptive statistics were presented in the mean and standard deviation for continuous data and frequency (absolute and relative) for categorical data. Differences in the demographic data between the groups were estimated using Student’s *t*-test (for age analysis) and Fisher’s exact test (for gender, smoking, and presence of other medication history analysis). Differences in the changes in clinical parameters between the two groups over the study periods were analyzed using repeated-measures ANOVA. Comparisons between the study periods within each group were done using Wilcoxon’s Signed Rank test and the changes at 4 and 8 weeks from the baseline between the groups were performed by the Mann-Whitney U test. A generalized estimating equation (GEE) analysis was used to assess the association between the treatment and various outcomes adjusting for confounding factors. Decreasing or increasing status on each outcome compared to the baseline value was the response variable and independent variables included age, gender, visit times and treatment group. Statistical significance was set at *p* < 0.05.

## Results

**Fig. 1:**
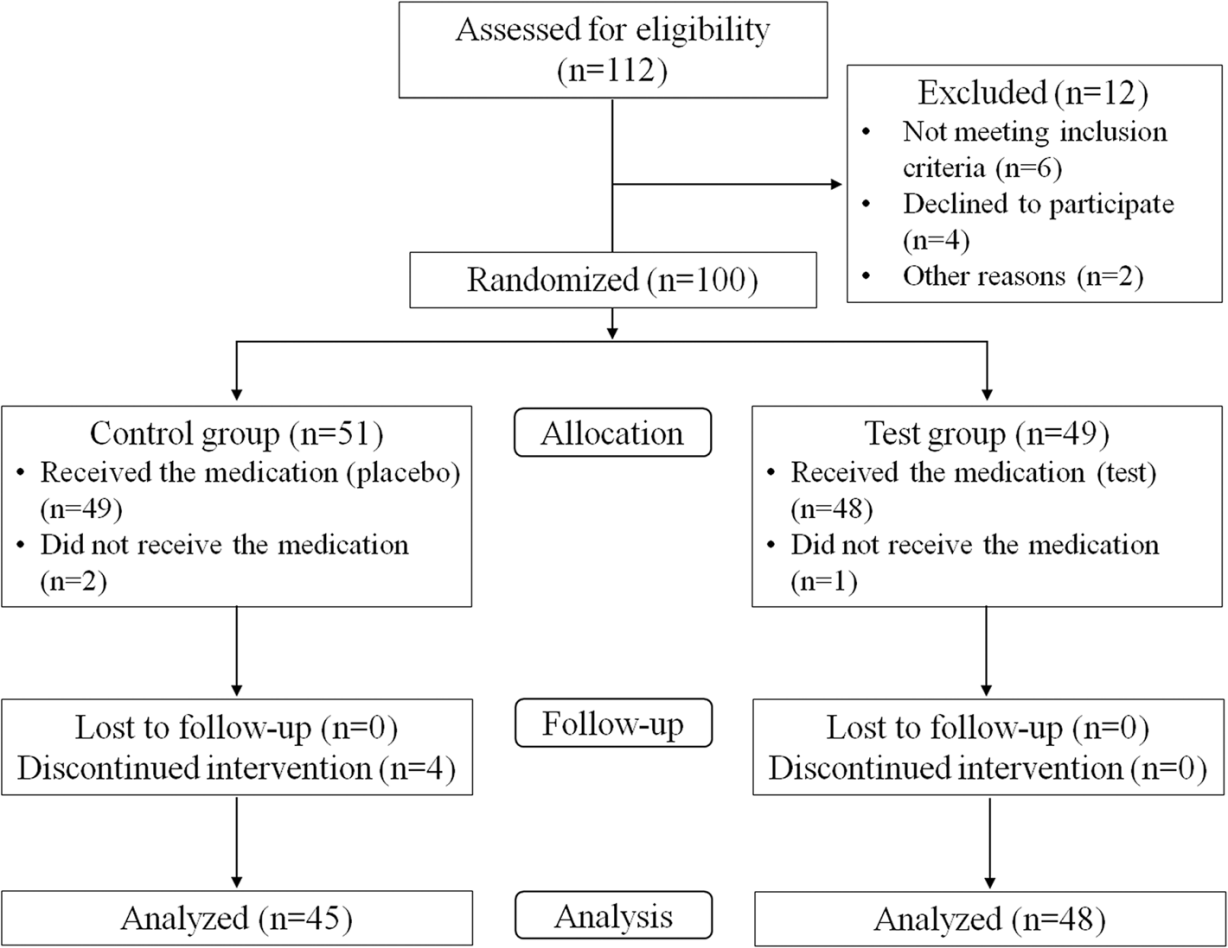
Flow chart of the clinical study

A total of 93 patients (45 in the control group and 48 in the test group) completed the study. The mean age was 43.02 ± 14.3 years in the control group and 37.83 ± 12.72 years in the test group. The percentage of female patients was 67.3% in the control group and 60.4% in the test group, and there were no significant differences in the gender distribution between the groups. The distribution of smoking status (nonsmoker, past smoker, and present smoker) and medication status for other systemic diseases between the groups also showed no significant differences (Table 1). Among the 100 patients initially enrolled in the study, seven were lost due to discontinued medication or intervention (Fig. 1). Throughout the intervention, there were no specific side effects, unintended effects or harms reported in each group.

**Table 1:** Demographic characteristics of the patients enrolled in the study

Variables	Control (*N* = 49)	Test (*N* = 48)	*p*-value
Age (years)	43.02 ± 14.30	37.83 ± 12.72	0.062
Gender			0.618
Male, *N* (%)	16 (32.7)	19 (39.6)	
Female, *N* (%)	33 (67.3)	29 (60.4)	
Smoking			0.578
Nonsmoker, *N* (%)	41 (83.7)	43 (89.6)	
Past smoker, *N* (%)	8 (16.3)	5 (10.4)	
Present smoker, *N* (%)	0 (0.0)	0 (3.3)	
Other medication history			0.660
No, *N* (%)	37 (75.5)	39 (81.2)	
Yes, *N* (%)	12 (24.5)	9 (18.8)	

*N*, number

Clinical parameters (GI, PI, PD, and CAL) and 100 mm VAS at baseline, 4 weeks and 8 weeks in the control and test groups are presented in Table 2. Differences in the changes in the parameters between the groups over 8 weeks were statistically significant only in the GI examinations (*p* = 0.042). For within group comparisons, the GI in the test group significantly decreased after 4 weeks (*p* < 0.001) and 8 weeks (*p* < 0.001) from the baseline, and the GI in control group showed no significant differences after 4 weeks and 8 weeks. The GI in the test group significantly decreased after 4 weeks from the baseline (Δ baseline – 4 weeks) compared with the control group (*p* = 0.015). In GEE model analysis adjusting for age, gender and visit times, the test group showed 2.5 times GI improvement compared to the control group (*p* = 0.022) (Table 3).

**Table 2:** Clinical parameters of the control and test groups at baseline, 4 weeks, and 8 weeks (mean ± SD)

Clinical parameters	Control (*N* = 45)	Test (*N* = 48)	*p*-value^*^
GI			0.042
Baseline	1.00 ± 0.46	1.19 ± 0.51	
4 weeks	1.01 ± 0.46	1.02 ± 0.44^†^	
8 weeks	0.90 ± 0.50	0.95 ± 0.49^†^	
Δ baseline – 4 weeks	0.01 ± 0.38	−0.18 ± 0.33^‡^	
Δ baseline – 8 weeks	−0.10 ± 0.40	−0.24 ± 0.38	
PI			0.138
Baseline	1.50 ± 0.68	1.61 ± 0.67	
4 weeks	1.45 ± 0.68	1.55 ± 0.58	
8 weeks	1.48 ± 0.61	1.42 ± 0.52^†^	
Δ baseline – 4 weeks	− 0.05 ± 0.40	−0.06 ± 0.48	
Δ baseline – 8 weeks	− 0.02 ± 0.39	−0.18 ± 0.52	
PD			0.381
Baseline	2.49 ± 0.39	2.63 ± 0.47	
4 weeks	2.47 ± 0.37	2.52 ± 0.49^†^	
8 weeks	2.39 ± 0.36	2.51 ± 0.51^†^	
Δ baseline – 4 weeks	− 0.02 ± 0.25	−0.11 ± 0.29	
Δ baseline – 8 weeks	− 0.10 ± 0.36	−0.11 ± 0.34	
CAL			0.571
Baseline	2.74 ± 0.69	2.76 ± 0.87	
4 weeks	2.75 ± 0.66	2.69 ± 0.84	
8 weeks	2.72 ± 0.71	2.72 ± 0.88	
Δ baseline – 4 weeks	0.01 ± 0.31	−0.07 ± 0.42	
Δ baseline – 8 weeks	− 0.02 ± 0.46	−0.03 ± 0.48	
100 mm VAS			0.059
Baseline	17.38 ± 16.69	18.46 ± 18.86	
4 weeks	13.42 ± 18.72^†^	11.17 ± 13.68^†^	
8 weeks	12.64 ± 16.90^†^	4.94 ± 6.34^†‡^	
Δ baseline – 4 weeks	−3.96 ± 21.45	−7.29 ± 17.55	
Δ baseline – 8 weeks	−4.73 ± 19.80	−13.52 ± 17.71	

*GI* gingival index, *PI* plaque index, *PD* probing depth, *CAL* clinical attachment level, *VAS* visual analog scale

^*^Difference in the change of each parameter during 8 weeks between the groups using repeated measures ANOVA (*p* < 0.05)

^†^Statistically significant difference compared to the baseline within each group using Wilcoxon Signed Ranks Test (*p* < 0.05)

^‡^Statistically significant difference between the groups using Mann-Whitney U test (*p* < 0.05)

**Table 3:** Generalized estimating equations for decreasing status of various endpoints

	β-estimate	SE	Odds ratio	*p*-value
GI (*N* = 97)				
Intercept	0.505	0.744	–	0.497
Group (reference; control)	0.899	0.394	2.457	0.022^†^
Visits (reference; week 4)	− 0.047	0.206	0.954	0.819
Gender (reference; male)	−0.246	0.383	0.782	0.521
Age	−0.010	0.015	0.990	0.479
PI (*N* = 97)				
Intercept	−0.092	0.540	–	0.864
Group (reference; control)	0.337	0.300	1.401	0.261
Visits (reference; week 4)	0.308	0.294	1.360	0.296
Gender (reference; male)	0.085	0.312	1.089	0.784
Age	−0.002	0.011	0.998	0.867
PD (*N* = 97)				
Intercept	0.277	0.599	–	0.644
Group (reference; control)	0.387	0.340	1.473	0.255
Visits (reference; week 4)	0.134	0.257	1.144	0.601
Gender (reference; male)	−0.372	0.346	0.689	0.283
Age	0.000	0.012	1.000	0.983
CAL (*N* = 97)				
Intercept	0.835	0.674	–	0.215
Group (reference; control)	0.169	0.359	1.184	0.638
Visits (reference; week 4)	−0.309	0.228	0.734	0.175
Gender (reference; male)	−0.386	0.364	0.680	0.289
Age	−0.008	0.013	0.992	0.524
100 mm VAS (*N* = 97)				
Intercept	−0.971	0.779	–	0.213
Group (reference; control)	0.364	0.408	1.440	0.372
Visits (reference; week 4)	0.488	0.202	1.629	0.016^†^
Gender (reference; male)	0.475	0.423	1.609	0.261
Age	0.021	0.016	1.021	0.192

*GI* gingival index, *PI* plaque index, *PD* probing depth, *CAL* clinical attachment level, *VAS* visual analog scale

^†^Statistically significant difference using GEE method (*p* < 0.05)

For within group comparisons, PI at 8 weeks (*p* = 0.045) and PD at 4 (*p* = 0.022) and 8 weeks (*p* = 0.018) in the test group significantly decreased from baseline. However, there were no significant differences for PI or PD compared with the control group. Additionally, 100 mm VAS in the control group significantly decreased after 4 weeks (*p* = 0.010) and 8 weeks (*p* = 0.039), and VAS in the test group also significantly decreased after 4 weeks (*p* = 0.004) and 8 weeks (*p* < 0.001). In comparing the 100 mm VAS between the groups, the test group significantly decreased after 8 weeks (*p* = 0.027). However, there was no significant difference in reduction in 100 mm VAS between groups in the GEE model.

## Discussion

Mechanical removal of subgingival plaque and debridement of the root surface have been the traditional and gold standard methods to control periodontal disease [18]. However, there are some cases where patients do not respond well to the treatment and exhibit a high susceptibility to disease. Dietary intake of micronutrients such as vitamins and minerals adjunctive to periodontal therapy has been expected to help maintain a balanced immune system by affecting several biological processes in the host response and enhancing innate immunity [9, 14]. In this sense, CELC in the present clinical intervention was evaluated for its effects on gingival inflammation and other periodontal parameters compared to a control group in chronic periodontitis patients.

Vitamin C, a water-soluble reducing agent that donates electrons, has been reported to maintain balanced redox potential of cells and scavenging ROS resulting from oxidative stress and downstream inflammatory responses [13, 14]. It also promotes the synthesis of normal mature collagen and intercellular material, wound healing, and host resistance to infection, all of which can cause gingival redness and swelling attributed to blood vessel damage [19]. Another nonenzymatic antioxidant, vitamin E, is a fat-soluble agent present in all cell membranes, which inhibits oxidative damage in membrane lipids [10]. It exhibits anti-inflammatory properties by reducing PGE_2_ production from macrophages and improving the humoral immune response [20–22]. Considering their potential roles in the inflammatory process, both vitamins have been investigated for their complementary use in gingivitis and periodontitis patients.

Previous studies utilizing serum biomarkers have demonstrated inverse associations between vitamin C, α-tocopherol (vitamin E) and total antioxidant level, and the prevalence of periodontitis, even though the findings were inconsistent and must undergo further evaluations [13, 23]. In a systematic review of clinical interventions, taking capsules containing each vitamin concentrate or customized dietary intake along with SRP showed conflicting results in their effects on the periodontal parameters, including probe depth, clinical attachment level, and bleeding index [11, 24]. A small or no significant clinical improvement in the adjunctive use of vitamin E or vitamin C was shown when applied as a single component, despite the benefits in the serum marker levels of antioxidant capacity. Since many ROS were formed in the aqueous phase, vitamin E may have limited actions as an antioxidant compared to vitamin C due to its lack of water-solubility and limited mobility confined to the cell membranes [25]. However, synergistic events might be expected when vitamin C is combined, as it has been shown to reduce vitamin E radicals created after scavenging oxygen radicals. The interactions between these two vitamins took place in both the homogenous aqueous solution and liposomal membrane environments, which may provide evidence of the advantages for the mixed use of vitamins C and E.

The results in the present study showed significant improvement in the mean change of GI within the first 4 weeks of the test group compared to the control group. The test group showed significant reduction in GI at both 4 weeks and 8 weeks from the baseline, whereas the control group showed no significant difference. Additionally, the test group showed approximately 2.5 times improvement (odds ratio 2.457, *p* = 0.022) compared to the control group when the confounding factors including age, gender, and visits were adjusted. However, the comparison of GI at each time point failed to show a significant difference between the groups. Other periodontal parameters including PI, PD and CAL did not show any significant differences in the test group when compared to the control group, although PI at 8 weeks and PD at 4 weeks and 8 weeks in the test group showed significant reduction from the baseline value. It can be assumed that these comparable outcomes are due to the SRPs done equally for both groups as the mechanical removal of plaque has effects on the improvement of PI, PD and CAL. Adjunctively supplemented CELC with SRP might have benefits for the reduction of superficial gingival inflammation, but the clinical effect did not reach the soft tissue status around the pocket base to change PD and CAL. Furthermore, it is still difficult to assert CELC’s clinical impact on gingival inflammation with a clear-cut conclusion as to the amount of mean change was very limited. To clarify its clinical efficacy, data from a larger sample size with full mouth examinations and longer study periods should further be obtained. Since the target subjects for adjunctive pharmaceuticals might include the ones with higher disease susceptibility, strict baseline criteria to enroll patients with severe periodontal disease should also be performed.

There has been little information on the clinical application of the nutritional uptake of lysozyme or carbazochrome for periodontal disease. In previous studies, host protective enzymes including lysozyme, lactoperoxidase, and lactoferrin were added to commercially available oral health care products such as toothpaste and mouth rinse to enhance saliva’s antimicrobial capacity [15]. Clinical trials on these products have been evaluated for their effects on the prevention of plaque accumulation, gingivitis, and dental caries with controversial results [15, 26]. However, there was a rough estimate that the reduction in plaque and gingivitis was approximately 10–20% within the limitations of various study designs and sample subjects [15]. Although direct comparisons of the clinical effects were not allowed due to the different study designs, methods of administration, and different compositions in the mixture, the addition of lysozyme and carbazochrome were expected to regulate the clinical symptoms of gingival bleeding and swelling in an indirect and synergistic way to help relieve the patient’s discomfort associated with gingival inflammation.

The control group received a placebo intervention provided capsules with a similar appearance to the medication in the treatment group but without essential components for the first 4 weeks and then was replaced by the treatment group medication for the following 4 weeks. The results showed a significant reduction in patient-reported 100 mm VAS at 4 and 8 weeks compared to the baseline in both groups. A significant difference in the intergroup comparison was seen at 8 weeks, but the reduction effect in VAS between the groups was not significant in the GEE model. A belief that placebo intervention substantially improves both patient-reported and observer-reported outcomes has been widely publicized with a variety of clinical conditions. However, the effects should be carefully analyzed as there has been a lack of comparison to the natural course of the disease (no treatment group) and the possibility of misinterpretation [27]. The systematic review evaluating the placebo intervention in various clinical conditions has demonstrated that it was difficult to find the important clinical effects, but the possible beneficial effects on patient-reported outcomes, especially pain, were observed in certain settings with placebo intervention [28]. Indistinguishable biased reporting and a wide range of standard deviations in the VAS data in the present study may drop the confidence in the improvements in results in both groups. Nevertheless, patient satisfaction with reduced gum discomfort and the motivations to achieve periodontal health could be acquired with adjunctive administration of CELC.

The patients enrolled in the present study included those who had PD values of 4–6 mm at at least one site for a quadrant and were diagnosed with chronic incipient to moderate periodontitis. However, the baseline data on PD in the control and test groups was 2.49 ± 0.39 mm and 2.63 ± 0.47 mm, respectively, of which the severity of periodontal disease might be considered to be in a mild state. The discrepancy in periodontal status may be due to the selected recordings of the Ramfjord teeth (teeth numbers 16, 21, 24, 36, 41, and 44) used in this study. Although a full mouth examination is considered the gold standard, there have been reports showing high agreement between Ramfjord teeth and full mouth periodontal probing that validated the partial recording in an epidemiological study [29, 30]. This technique might be more effective in handling a large sample size, but controversial opinions have demonstrated lower intraclass correlation coefficients for Ramfjord teeth assessments for the percentage of sites over a higher threshold and underestimation of disease prevalence [31]. The partial recording system of selected teeth in the present study might have limited the precise interpretations of the changes in the clinical results.

Within the limitations of the present study, CELC adjunctively administered SRP exhibited a significant reduction in the index of gingival inflammation in a short-term investigation. Patient’s self-reported gingival discomfort improved in both groups with significant differences at 8 weeks between the two groups. However, changes in other periodontal parameters, including PI, PD and CAL, were similar between the groups. CELC’s effect on the reduction in gingival inflammation should further be clarified with larger sample sizes and clinical data from full mouth examinations. Additionally, studies on adjunctive nutritional intake for compromised populations such as the elderly or comorbidities should be further evaluated.

## Conclusions

Within the limitations of the study, adjunctively supplemented CELC with SRP showed a significant reduction in gingival inflammation compared with a placebo in a short-term investigation. However, other periodontal parameters were similar between groups, and CELC’s clinical efficacy and benefits should further be clarified with a larger sample size.

## Abbreviations

AP-1: Activating protein-1
CAL: Clinical attachment level
GE: Gingival enlargement
GI: Gingival index
GR: Gingival recession
IL-1: Interleukin-1
NF-κB: Nuclear factor kappa B
PD: Probing depth
PI: Plaque index
PP: Per protocol
ROS: Reactive oxygen species
SRP: Scaling and root planning
TEAEs: Treatment-emergent adverse events
VAS: Visual analogue scale
